# Biocontrol endophytes *Bacillus subtilis* R31 influence the quality, transcriptome and metabolome of sweet corn

**DOI:** 10.7717/peerj.14967

**Published:** 2023-03-02

**Authors:** Mingwei Shao, Yanhong Chen, Qingyou Gong, Shuang Miao, Chunji Li, Yunhao Sun, Di Qin, Xiaojian Guo, Xun Yan, Ping Cheng, Guohui Yu

**Affiliations:** 1 College of Agriculture and Biology, Zhongkai University of Agriculture and Engineering, Guangzhou, China; 2 Innovative Institute for Plant Health, Zhongkai University of Agriculture and Engineering, Guangzhou, China; 3 Key Laboratory of Green Prevention and Control on Fruits and Vegetables in South China, Ministry of Agriculture and Rural Affairs, Guanghzou, China; 4 Guangdong University Key Laboratory for Sustainable Control of Fruit and Vegetable Diseases and Pests, Guangdong, China; 5 Zhuhai Modern Agriculture Development Center, Zhuhai, China

**Keywords:** Biocontrol bacterium, *Bacillus subtilis* R31, Sweet corn, Metabolomics, Transcriptomics

## Abstract

During colonization of soil and plants, biocontrol bacteria can effectively regulate the physiological metabolism of plants and induce disease resistance. To illustrate the influence of *Bacillus subtilis* R31 on the quality, transcriptome and metabolome of sweet corn, field studies were conducted at a corn experimental base in Zhuhai City. The results show that, after application of *B. subtilis* R31, sweet corn was more fruitful, with a 18.3 cm ear length, 5.0 cm ear diameter, 0.4 bald head, 403.9 g fresh weight of single bud, 272.0 g net weight of single ear, and 16.5 kernels sweetness. Combined transcriptomic and metabolomic analyses indicate that differentially expressed genes related to plant-pathogen interactions, MAPK signaling pathway-plant, phenylpropanoid biosynthesis, and flavonoid biosynthesis were significantly enriched. Moreover, the 110 upregulated DAMs were mainly involved in the flavonoid biosynthesis and flavone and flavonol biosynthesis pathways. Our study provides a foundation for investigating the molecular mechanisms by which biocontrol bacteria enhance crop nutrition and taste through biological means or genetic engineering at the molecular level.

## Introduction

Microbial biotechnology plays an irreplaceable role in many aspects, such as environmental remediation and protection, renewable energy production, and human health protection (Chen et al., 2007). The diversity of physiological metabolism and life strategies of microorganisms provides an important guarantee for humans to seek solutions to environmental, energy, and health problems (Eida et al., 2020; Zhou et al., 2021). Biological control has many advantages over chemical control, including the safety of humans, animals, and natural enemies (Arnaouteli et al., 2021). Meanwhile, it has good compatibility with the environment and can control harmful organisms in a long-term and stable manner (Andri, Meyer & Ongena, 2020). It has become the focus of research on environmental protection, low resistance, and strong selectivity in plant disease control (Duy et al., 2007; Fu et al., 2021; Kuchina et al., 2021) and has been used in agricultural production and has achieved good social and economic benefits (Otto et al., 2010; Nicolas et al., 2012). For example, in Alabama, scientists treated crop seeds with *Bacillus subtilis* and found that it significantly increased the average yields and effectively prevented crop diseases. Studies have shown that biocides can effectively control Asian soybean rust disease in Brazil, improving plant resistance, promoting plant growth, and controlling pests. Japanese scientists have also found that *B. subtilis* has a good control effect on tomato blight (Arnaouteli et al., 2021).

Biological control mainly utilizes growth-promoting effects, hyperparasitism, antibiosis, lysis, competition, predation, or cross-protection, and other forms to reduce the number of pathogens or weaken their pathogenicity to change the relationship between plants and pathogens, and inhibit the occurrence of related diseases (Qin et al., 2019). At present, there are mainly two types of microbial biological control measures for plant diseases: one is to introduce exogenous antagonistic bacteria into crops or soil, and the other is to regulate the environmental microbial community of plant growth so that the existing beneficial microbial community can increase and show antagonistic activity (Meng et al., 2018; Futo et al., 2021). Some biocontrol bacteria can produce antibacterial substances and effectively inhibit the growth of pathogenic bacteria, which is called the antibacterial effect (Seedorf et al., 2008; Li et al., 2021a). Studies have shown a close relationship between plants and microorganisms in the soil (Wu et al., 2018). However, studies on the transcriptome and metabolome of plants subjected to biocontrol bacteria are relatively rare (Cutulle et al., 2018).

The endophytic *B. subtilis* R31 isolated from the leaves of *Dendrobium orchid* is a typical example of an exogenous endophyte for controlling banana fusarium wilt in China (Li et al., 2021a). The strain and its patent have been industrialized by Guangdong Geolong Biotechnology Co., Ltd. “*B. subtilis* R31” received a Chinese common name from the National Pesticide Standardization Technical Committee, and the trade name is “Dingwei”. *B. subtilis* R31 has been popularized and applied in major banana-producing areas such as Yunnan and Hainan, and excellent economic benefits have been achieved in the prevention of fusarium wilt of susceptible banana varieties in newly planted banana gardens since 2019. To further expand the application range of *B. subtilis* R31, we analyzed the quality of sweet corn after the application of R31. Here, we analyzed ear length, ear diameter, bald head, fresh weight of a single bud, net weight of a single ear, and kernel sweetness of sweet corn treated with *B. subtilis* R31 in the field. Based on the above studies, we systematically analyzed the influence of the transcriptome and metabolome of sweet corn after applying *B. subtilis* R31, which provide a foundation for the prevention of crop diseases and the promotion of production and quality of crop.

## Materials and Methods

### Plant materials and culture conditions

The isolation and identification of *B. subtilis* R31 have been described in detail in a previous study (Li et al., 2021a). Here, we briefly describe the preparation process of the bacterial suspension. A single colony was picked and inoculated into 5 mL of LB solution at 37 °C and 180 rpm for 8 h. One hundred microliters of seed solution was inoculated into 100 mL of LB solution and incubated at 37 °C and 180 rpm for 8 h. The bacteria were collected by centrifugation at 10,000 rpm and resuspended in 100 mL sterile water to wash off the residual medium. The procedure was repeated twice, and the bacteria were suspended at a concentration of approximately 1 * 10^8^cfu in sterile water for field experiments.

The field trial was conducted in October 2020 at the Corn Experimental Base of Taiwan Farmers’ Pioneer Park in Jinwan, Zhuhai City (111°22′ N, 23°9′ E). A random block test was adopted in the experiment, and each treatment was repeated in three groups with 30 plants in each block. Zhuyutian No. 1 seeds, a variety of sweet corn, were sown in a plug tray with matrix soil for 10 d to raise the seedlings. Seedlings were transplanted to field at 3–4 leaf stage. At the same time, each seedling was watered with 100 mL of the biocontrol suspension, and the control was filled with 100 mL of water. The bacterial suspension was then watered each month and four times throughout the growth cycle. Twenty days after the last watering, the record of ear length, ear diameter, bald head, fresh weight of single bud, net weight of single ear, and kernel sweetness of sweet corn were performed. Three pieces of corn on the cob were then randomly selected, and the bracts were removed. The grains were cut off with a knife disinfected with alcohol, and 30 grains were randomly selected. Selected grains were placed in sterile tubes and stored in liquid nitrogen. The prepared samples were sent to MetWare Biological Science and Technology Co. Ltd. (Wuhan, China) for transcriptome sequencing on an Illumina HiSeq 2500 platform and for widely targeted metabolome analysis (Li et al., 2012, 2021b).

### Transcriptome sequencing and data analysis

Transcriptome analyses of sweet corn under the influence of the biocontrol *B. subtilis* R31 were conducted. Sweet corn with or without treatment with *B. subtilis* R31 were collected in three biological duplications for each treatment, which were marked as CK and R31. The concentration, quality, and integrity of the isolated RNA were checked using a NanoDrop spectrophotometer (Thermo Fisher Scientific, Waltham, MA, USA), agarose gel electrophoresis, Qubit 2.0 fluorometer (Qubit, Dorset, UK), and Agilent 2100 bioanalyzer (Agilent, Santa Clara, CA, USA). Qualified RNA samples were subjected to transcriptome sequencing. The statistical power of this experimental design was calculated in RNASeqPower is 0.63. Raw data were filtered using FASTP V 0.19.3, which mainly removes reads with adapters. Reads with adapters, including sequences with more than 10% unknown nucleotides (N) and more than 50% low-quality sequences (quality score less than Q20), were removed. Transcriptomic sequencing analysis of six samples was completed in this study, and all subsequent analyses were based on the percentage of Q30 base more than 93%. Gene expression levels were analyzed based on the results of sequencing data comparison. Differentially expressed genes (DEGs) were identified based on their expression levels in CK and R31. As well as the functional annotation and enrichment analyses were performed. All clean reads were sequenced with the reference genome using HISAT2 to obtain the location information of the reference genome or genes as well as the sequence characteristic information of the sequenced samples. The reads were assembled into transcripts using StringTie, based on the location information of the reads compared to the reported genome. The spliced transcripts were then compared with annotated genome information using GffCompare software. Then, the target gene sequences were extracted from the genome and compared with the Kyoto Encyclopedia of Genes and Genomes (KEGG), GO, NR, Swiss-Prot, trEMBL, and KOG databases using the BLAST software to obtain the annotated results.

Fragments per kilobase of transcript per million fragments mapped (FPKM) were used to measure the transcription or gene expression level. DESeq2 was used to analyze the expression of DEGs in biological duplicates, with a Pearson’s correlation coefficient (R) greater than 0.8. Principal component analysis (PCA) was performed on the regularized log values of the read counts. Next, the Benjamini-Hochberg method was used to conduct multiple hypothesis testing correction for hypothesis testing probability (*p*-value) to obtain the false discovery rate (FDR). DEGs were obtained with the screening conditions of | log_2_Fold change |≥ 1 and FDR <0.05. The GO and KEGG pathway and enrichment analyses of detected DEGs were performed to illustrate how the transcriptome of sweet corn responds to biocontrol *B. subtilis* R31.

### Widely targeted metabolome analysis

Metabolomic analyses of sweet corn treated with the biocontrol *B. subtilis* R31 were conducted. Frozen sweet corn samples were placed in a lyophilizer (ScientZ-100F), vacuum freeze-dried, and ground to powder with a grinder (MM 400, Retsch, Haan, Germany) (30 Hz, 1.5 min). The 100 mg powder was dissolved in 1.2 mL 70% methanol and was extracted by vortex every 30 min, each time lasting 30 s and repeated six times. The sample was then placed in a refrigerator at 4 °C overnight, centrifuged at 12,000 rpm for 10 min, and the supernatant was collected. Subsequently, the samples were filtered through a microporous membrane (0.22 μm pore size) and stored in an injection vial for UPLC-MS/MS analysis. To ensure the accuracy of the assay, six samples were mixed as quality control (QC) samples and ultra-performance liquid chromatography and tandem mass spectrometry were included in the data acquisition instrument system. HPLC analyses were performed using Agilent 1260 Infinity equipment with a diode array detector (DAD) (Agilent Technologies, Santa Clara, CA, USA) equipped with a reversed-phase column (Agilent SB-C18 1.8 µm, 2.1 * 100 mm) with a column temperature of 40 °C. Phase A was ultrapure water (0.1% formic acid), and phase B was acetonitrile (0.1% formic acid). The liquid elution gradient was as follow: 0 min B phase proportion was 5%, within 9 min the B phase proportion increased linearly to 95% and was maintained at 95% for 1 min; 10.00–11.10 min, B phase proportion decreased to 5% and was balanced at 5% to 14 min. The flow rate was set at 0.35 mL/min, and the injection volume was 4 μL. LIT and triple quadrupole (QQQ) scans were acquired using a triple quadrupole linear ion trap mass spectrometer (Q TRAP) AB4500 Q TRAP UPLC/MS/MS system. The system was equipped with an ESI Turbo ion spray interface, which can be controlled by the Analyst 1.6.3 software (AB Sciex, Framingham, MA, USA) to run both positive and negative ion modes. The ESI source operating parameters were as follows: ion source, turbo spray; source temperature, 550 °C; ion spray voltage (IS), 5,500 V (positive ion mode)/−4,500 V (negative ion mode); ion source gas I (GSI), gas II (GSII), and curtain gas (CUR) were set to 50, 60, and 25.0 psi, respectively, and the collision-induced ionization parameters were set to high. QQQ scans used the multiple reaction monitoring (MRM) mode and set the collision gas (nitrogen) to the medium. The collision energy (CE) and declustering potential (DP) of each MRM transition were determined by further optimization. A specific set of MRM transitions was monitored in each epoch, based on the metabolites eluted.

The material was characterized according to the MS/MS spectrum information based on the MWDB (MetWare Biological Science and Technology Company, Ltd., Woburn, MA, USA). Isotope signals, repeated signals containing K^+^, Na^+^, NH_4_^+^, and fragments of other substances with larger molecular weights were removed during the analysis. Software Analyst 1.6.3 was used to process the mass spectrum data. PCA was used to summarize the characteristics of the subjects’ metabolic profiles. PCA presents unit variance scaling (UV) normalization for data using the built-in statistical prcomp function of R software (www.r-project.org/), which was used to set the prcomp function parameter scale = True. The metabolite content data were normalized (unit variance scaling and UV scaling), and heat maps were drawn using the R software pheatmap package. Hierarchical cluster analysis (HCA) was conducted to determine the accumulation patterns of metabolites in the different samples. Differentially accumulated metabolites (DAMs) between CK and R31 were determined using variable importance in projection (VIP) ≥1 and absolute Log_2_FC (fold change) ≥1. VIP values were extracted from the results tested using the OPLS-DA model as described by Wang et al. (2019) and Shang et al. (2021). GO and KEGG pathway and enrichment analyses of the detected DAMs were performed to illustrate the influence of the transcriptome of sweet corn responsive to biocontrol *B. subtilis* R31.

## Results

### Biocontrol *Bacillus subtilis* R31 improves sweet corn quality

**Figure 1:**
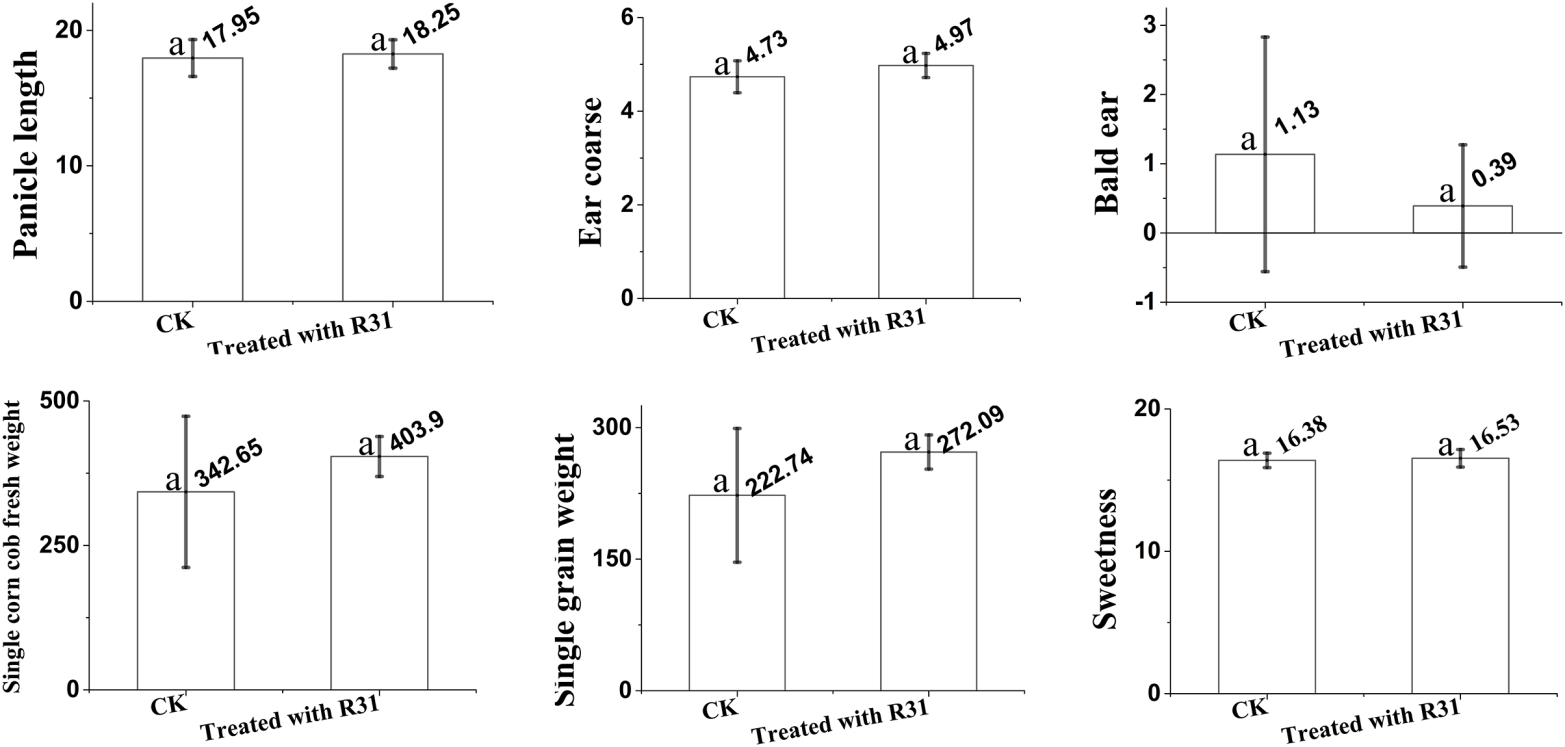
Overview of analysis of *Bacillus subtilis* R31 improves the quality of sweet corn. Changes in maize after root irrigation control and treatment after application of *B. subtilis* R31. Analysis of variance (ANOVA) was performed on maize for ears length, ears thickness, baldness, fresh weight, net weight and sweetness. The SD (standard deviation) of the means was calculated from 11 replicates and analyzed using a one-way ANOVA (*p* ≤ 0.05). Letters at the top of each bar (a) represent differences, with a indicating the highest. Identical letters indicate that no significant differences were observed.

To further expand the application range of *B. subtilis* R31, we analyzed the quality of sweet corn after the application of R31. Here, we analyzed ear length, ear diameter, bald head, fresh weight of a single bud, net weight of a single ear, and kernel sweetness of sweet corn treated with *B. subtilis* R31 in the field. The results (Fig. 1) indicate that, after application of *B. subtilis* R31, sweet corn was more fruitful with an 18.3 cm ear length, 5.0 cm ear diameter, 0.4 bald head, 403.9 g fresh weight of single bud, 272.0 g net weight of single ear, and 16.5 kernels sweetness than untreated corn, with an 18.0 cm ear length, 4.7 cm ear diameter, 1.1 bald head, 342.7 g fresh weight of single bud, 222.7 g net weight of single ear, and 16.4 kernels sweetness.

### Illumina sequencing and assembly of functional annotation of unigenes

Three independent replicates of sweet corn responsive to biocontrol *B. subtilis* R31 (CK and R31-T) were subjected to RNA-Seq analyses to demonstrate the influence on the transcriptome and metabolome of sweet corn. A total of six libraries of CK and R31 resulted in approximately 7.45−8.41 Gb clean reads, a ratio of fuzzy bases (N) less than 0.03%, a Q20 value greater than 97.7%, and a Q30 value greater than 93.6% (Table S1). Alignment efficiency refers to the percentage of mapped reads in the clean reads. It was more than 70% of the total with a fully equipped assembly, matched well with the reference genome, and was without contamination. After assembling the clean reads, an alignment efficiency of >84.0% was observed (Table S1 and Fig. S1). The proportion of reads located in the exon region was more than 86.0% (Fig. S2). The unigenes obtained from the NR annotation were aligned against other species, and 97.38% of the unigenes were mapped to *Zea mays* (Fig. S3), which was largely consistent with a previous report (Hu et al., 2021). Overall, the data indicate that sequencing was of high quality and met the requirements for further analysis.

**Figure 2:**
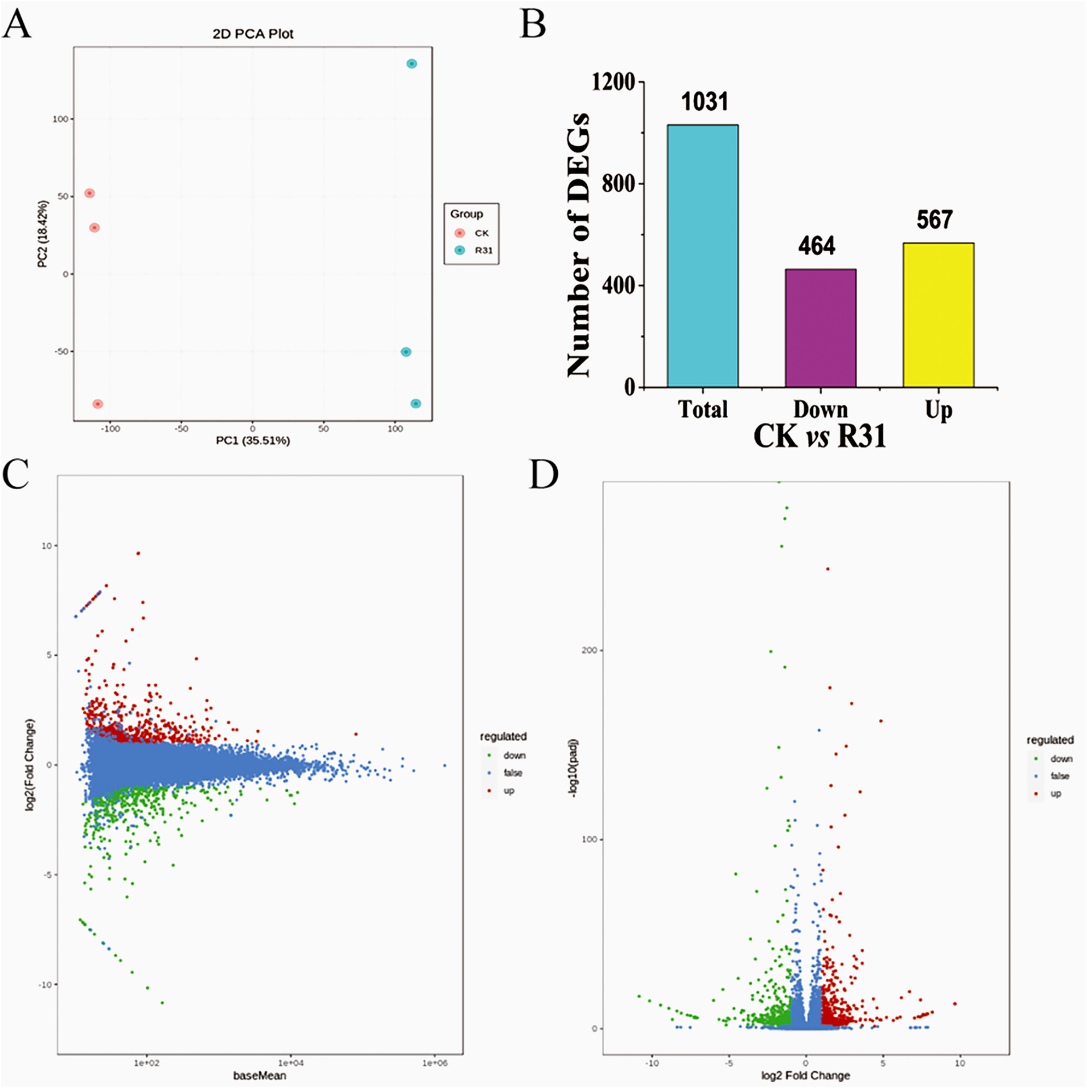
Overview of transcriptome analysis of sweet corn after application of *B. subtilis* R31. (A) PCA analysis of the expression of unigenes for CK *vs* R31. (B) Bar graph of up- and downregulated genes from pairwise comparisons. (C) MA plots for expression analysis contrasts between the CK and R31. The ordinate represents the log_2_ FC value, which represents the logarithm of the difference multiple (log_2_ fold change). The horizontal coordinate represents the average amount of gene expression in the two samples. Red dots represent up-regulated gene expression, green dots down-regulated gene expression, and blue dots show no significant difference in gene expression. (D) Volcano map of DEGs of CK *vs* R31. The horizontal coordinate indicated the change of gene expression multiple, and the vertical coordinate indicated the significance level of differential genes. The red dots represent up-regulated differential genes, the green dots represent down-regulated differential genes, and the blue dots represent non-differentially expressed genes.

All 331,174 unigenes were searched in the NR, GO, KEGG, Pfam, KOG, and Swiss-Prot databases, with 36,604, 28,977, 24,867, 29,349, 32,265, and 24,970 corresponding annotated unigenes, respectively (Tables S2–S8). The unigenes annotated in the eggKOG database were divided into 25 categories, of which “general function prediction only,” “posttranslational modification, protein turnover, chaperones,” and “signal transduction mechanisms” contained the most unigenes (Table S9 and Fig. S4). GO term analyses of the sweet corn stem transcriptome showed that 18 terms were related to the “cellular component,” 13 terms were correlated with the “molecular function” and 28 terms were included in “biological process” (Table S10). Altogether, 135 KEGG pathways were annotated. Results showed that the “signal transduction”, “carbohydrate metabolism,” and “translation” were relative enrichment (Table S11). Correlation evaluation of biological repetition for transcriptome analyses of sweet corn after application of *B. subtilis* R31 was conducted (Fig. 2A). The results demonstrated the high uniformity of the samples in a group, which provided high credibility for subsequent DEGs analysis. The PCA showed a sharp distinction between the CK and R31 was largely accounted for by PC1 (21.73%), whereas PC2 (21.27%) accounted for the difference between sweet corn varieties.

### Biocontrol *Bacillus subtilis* R31 influences the transcriptome of sweet corn

DEGs in different comparisons were identified using DESeq based on FPKM values. Comparison between CK and R31 yielded 1031 DEGs, 567 upregulated and 464 downregulated (Fig. 2B). This result indicates that there was an appreciable change in gene expression levels in sweet corn after the application of *B. subtilis* R31. In CK *vs*. R31, all DEGs were clustered into nine groups, presented as heatmaps (Fig. S6 and Table S12). Gene ontology of the DEGs showed that 14 terms were related to the “cellular component,” 9 terms were correlated with “molecular function” and 22 terms were included in “biological process” (Fig. S7 and Table S13). The main GO terms in the cellular component category were membrane (157 DEGs), cell (350 DEGs), organelle (261 DEGs), organelle part (76 DEGs), membrane part (126 DEGs), and cell part (348 DEGs). Catalytic activity (277 DEGs) and binding (316 DEGs) were the most abundant GO terms for “molecular function”. Metabolic process (280), cellular process (309 DEGs), regulation of biological process (141 DEGs), response to stimulus (173 DEGs), and biological regulation (155 DEGs) were the most abundant GO terms related to the biological process (Table S14). GO enrichment (GO enrichment top50) showed that the DEGs related to “biological process” were distributed in the terms defense response to fungus, cellular response to salicylic acid stimulus, cellular response to cold, response to organonitrogen compound, response to organic cyclic compound, response to jasmonic acid, response to fungus, response to ethylene, response to chitin, response to antibiotic, jasmonic acid-mediated signaling pathway, salicylic acid-mediated signaling pathway, and transposition. DEGs related to “cellular component” were distributed in the terms PRC1 complex, PcG protein complex, nuclear ubiquitin ligase complex, methyltransferase complex, histone methyltransferase complex, extracellular region part, ESC/E(Z) complex, and apoplast (Fig. S8). DEGs related to molecular function were distributed in the terms xyloglucosyl transferase activity, RNA-directed DNA polymerase activity, DNA polymerase activity, and aspartic-type peptidase activity.

**Figure 3:**
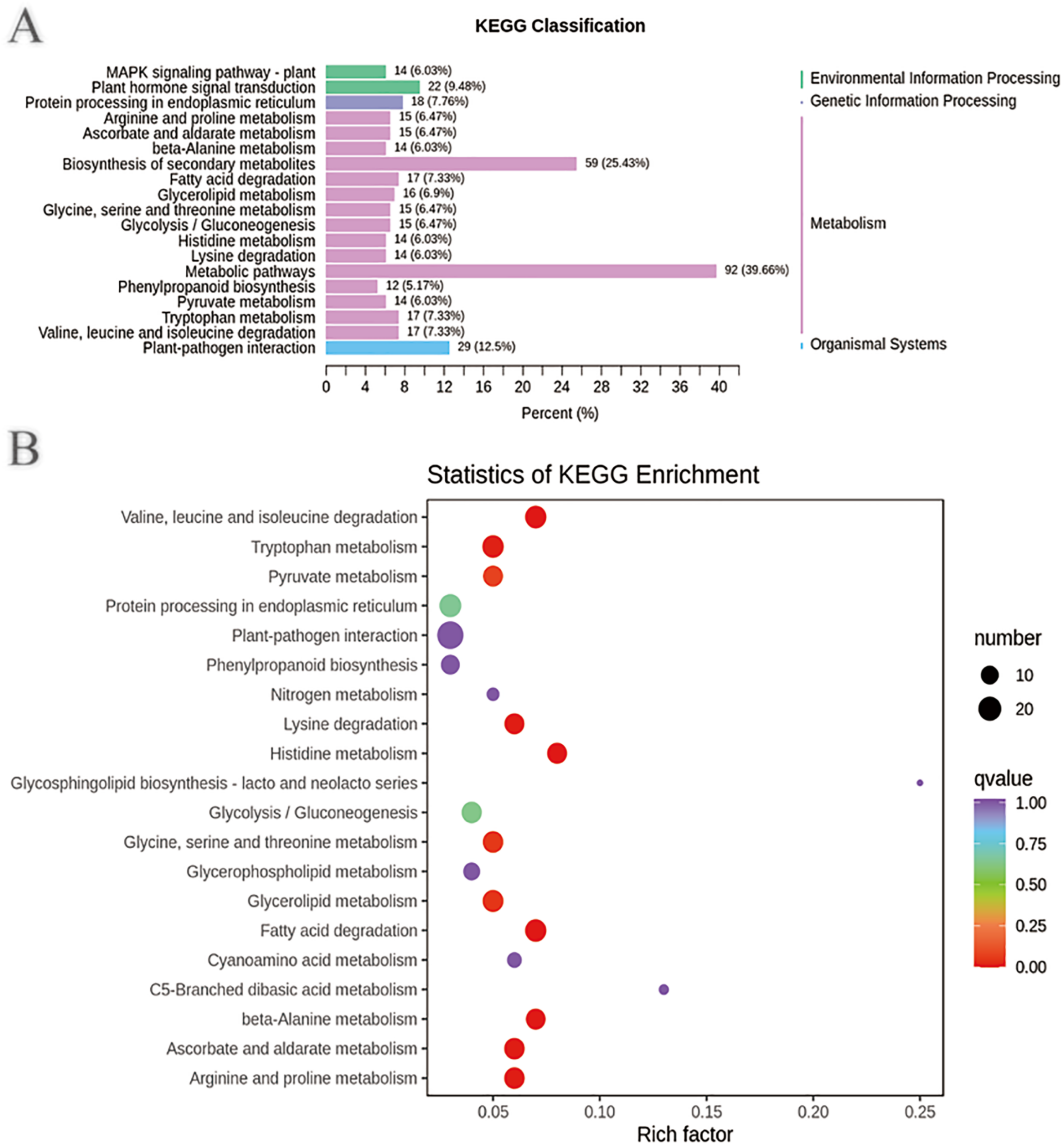
Enriched KEGG pathways of DEGs in sweet corn after application of *B. subtilis* R31. (A) Histogram of KEGG classification of DEGs for CK *vs* R31. The horizontal coordinate represents the ratio of genes annotated to the pathway to the total number of genes annotated, and the vertical coordinate represents the name of the KEGG pathway. The label to the right of the figure represents the classification to which the KEGG channel belong (B) KEGG enrichment map of DEGs for CK *vs* R31. The abscissa represents the rich factor corresponding to each pathway, and the ordinate represents the pathway name. The color of the points reflects the *p*-value value, and the red indicates the more significant enrichment. The size of the dots represents the number of differentiated metabolites enriched.

In addition, the DEGs annotated in the clusters of orthologous groups of proteins (COG) database were mainly distributed in the categories of posttranslational modification, protein turnover, chaperones (39), signal transduction mechanisms (26), intracellular trafficking, secretion, and vesicular transport (10), transcription (18), replication, recombination and repair (10), energy production and conversion (11), carbohydrate transport and metabolism (15), secondary metabolite biosynthesis, transport and catabolism (15), and general function prediction only (39) (Fig. S9 and Tables S15–S16). These DEGs may be involved in plant disease resistance and growth promotion affected by the biocontrol *B. subtilis* R31. KEGG pathway analysis of DEGs showed that there were 2, 1, 15, and 1 pathways classified into environmental information processing, genetic information processing, metabolism, and organismal systems, respectively (Fig. 3A and Table S17). Most DEGs were enriched in metabolic pathways (92), biosynthesis of secondary metabolites (59), plant-pathogen interaction (29), plant hormone signal transduction (22), tryptophan metabolism (17), glycolysis/gluconeogenesis (15), mitogen-activated protein kinase (MAPK) signaling pathway (14), and phenylpropanoid biosynthesis (12), which indicated that *B. subtilis* R31 affected the production of secondary metabolites in sweet corn, thus affecting its taste (Fig. 3B). The DEG-enriched KEGG pathways were distributed mostly in amino acid metabolism, such as the degradation of lysine, valine, leucine, and isoleucine, and the metabolism of histidine, glycine, serine, threonine, arginine, and proline. There were also DEGs related to pyruvate metabolism, glycerolipid metabolism, fatty acid degradation, beta-alanine metabolism, ascorbate, and aldarate metabolic pathways.

### Multivariate analysis of identified metabolites

**Figure 4:**
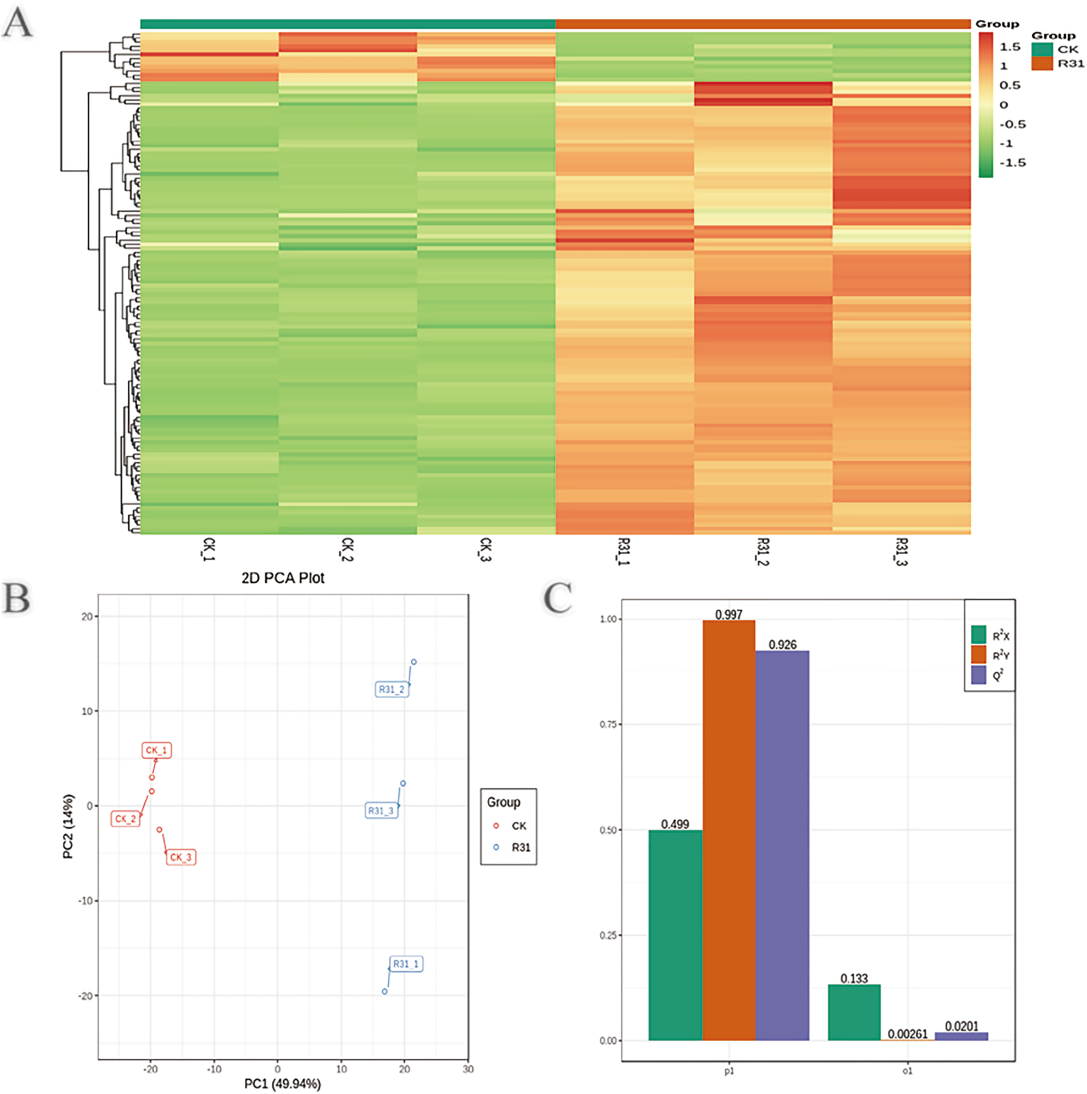
Overview of widely targeted metabolome analysis of sweet corn after application of *B. subtilis* R31. (A) Cluster analysis of metabolites from CK *vs* R31. Each example was visualized in a single column, and each metabolite is represented by a single row. The color indicates the level of accumulation of each metabolite, from low (green) to high (red). (B) PCA of metabolites for CK *vs* R31. (C) The OPLS-DA model of metabolites for CK *vs* R31.

**Figure 5:**
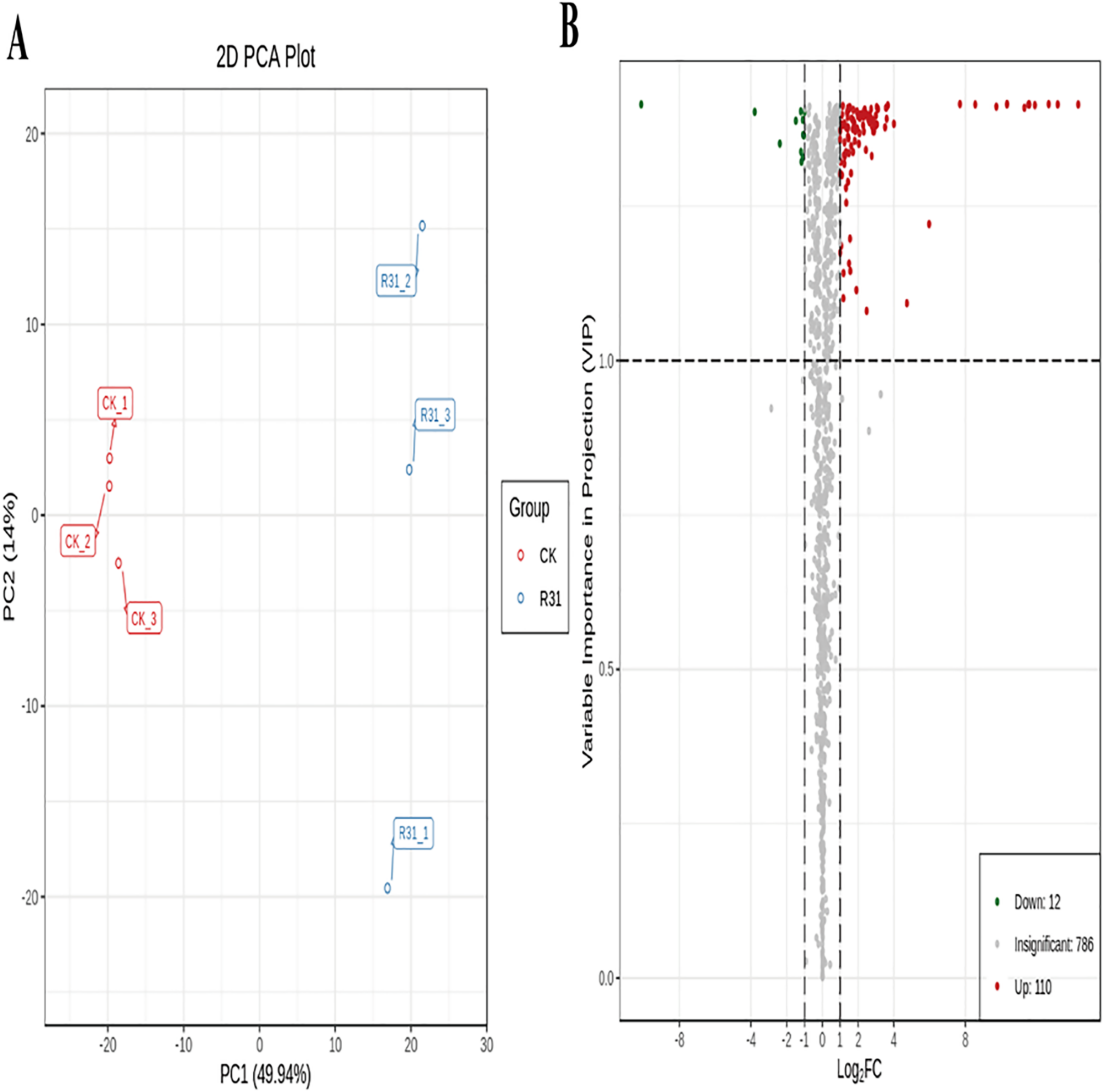
PCA and OPLS-DA of DAMs identified among sweet corn after application of *B. subtilis* R31. (A) Group principal component analysis diagram. (B) Volcano plot of metabolites. (A) Group principal component analysis diagram. (B) Volcano plot of metabolites. Each dot in the volcanic map represents metabolite. The green dots representing down-regulated differential metabolites, the red dots representing up-regulated differential metabolites, and the gray dots representing metabolites detected but not significantly different. The horizontal coordinate represents the logarithm (log_2_ FC) of the multiple of the relative content difference between the two groups of samples.

To detect the influence on the metabolome of sweet corn by biocontrol *B. subtilis* R31, the extracts from treated and untreated sweet corn were analyzed by MRM. In total, 908 metabolites were identified, which showed distinct hierarchical clustering of samples in the heatmap visualization (Fig. 4A and Table S18). PCA of the data set from the 908 metabolites was performed to examine all metabolomic differences among the three varieties. PCA clearly separated the three varieties and QC samples with a significance of 0.01, and the repeated samples were tightly clustered (Fig. 4B). Orthogonal signal correction was applied, and a partial least squares-discriminant analysis (OPLS-DA) model was used to determine the differences in metabolite composition, and Q^2^ exceeded 0.9 (R^2^X = 0.499, R^2^Y = 0.997, Q^2^ = 0.926) (Fig. 4C). The above results show that the entire analysis was stable and repeatable and could be used to further screen for DAMs. A total of 122 DAMs were obtained for the comparison of sweet corn response to biocontrol *B. subtilis* R31 (CK and R31-T), according to the quantitative analyses of all detected metabolites based on PCA and OPLS-DA analyses (Figs. 5A and S10). The results show that 12 metabolites were downregulated and 110 metabolites were upregulated in sweet corn treated with the biocontrol *B. subtilis* R31, which confirmed the significant differences in the heatmap of DAMs (Fig. 5B and Table S19).

### Biocontrol *Bacillus subtilis* R31 influences the metabolome of sweet corn

The DAMs in sweet corn responsive to biocontrol *B. subtilis* R31 were mainly classified into 10 categories: flavonoids (75), phenolic acids (16), lignans and coumarins (nine), alkaloids (six), organic acids (three), nucleotides and derivatives (four), amino acids and derivatives (one), terpenoids (two), lipids (one), and others (six) (Table S19). The levels of 110 metabolites were higher and of 12 metabolites were lower in R31 than in CK, and 122 DAMs were mainly secondary metabolites. In addition, 110 upregulated secondary metabolites comprising 72 flavonoids, 14 phenolic acids, two terpenoids, nine lignans and coumarins, one amino acid and derivatives, five alkaloids, three nucleotides and derivatives, one lipid, and three others, and 12 downregulated secondary metabolites comprising two flavonoids, one nucleotide and derivatives, two phenolic acids, three organic acids, one alkaloid, and three others were identified (Table S19). According to Log_2_FC (fold change) and VIP values, the majority of upregulated DAMs (Log_2_FC ≥3) were luteolin-6-C-glucoside (isoorientin), luteolin-8-C-glucoside (orientin), orientin-2″-O-galactoside, swertiajaponin, epigallocatechin-3-gallate, luteolin-7-O-(6″-sinapoyl) glucoside, diosmetin-7-O-neohesperidoside (neodiosmin), mandelic acid, luteolin-6-C-(2″-glucuronyl) glucoside, and 3,4,2′,4′,6′-pentahydroxychalcone; downregulated DAMs were caffeine and diosmetin-7-O-glucuronide, pinoresinol-4,4′-O-di-O-glucoside, chrysoeriol-6-C-rhamnoside-7-O-rhamnoside, L-ascorbic acid(vitamin C), 2-isopropylmalic acid, 4-methyl-5-thiazoleethanol, 5-methyluridine, and 3-isopropylmalic acid (Fig. S11).

**Figure 6:**
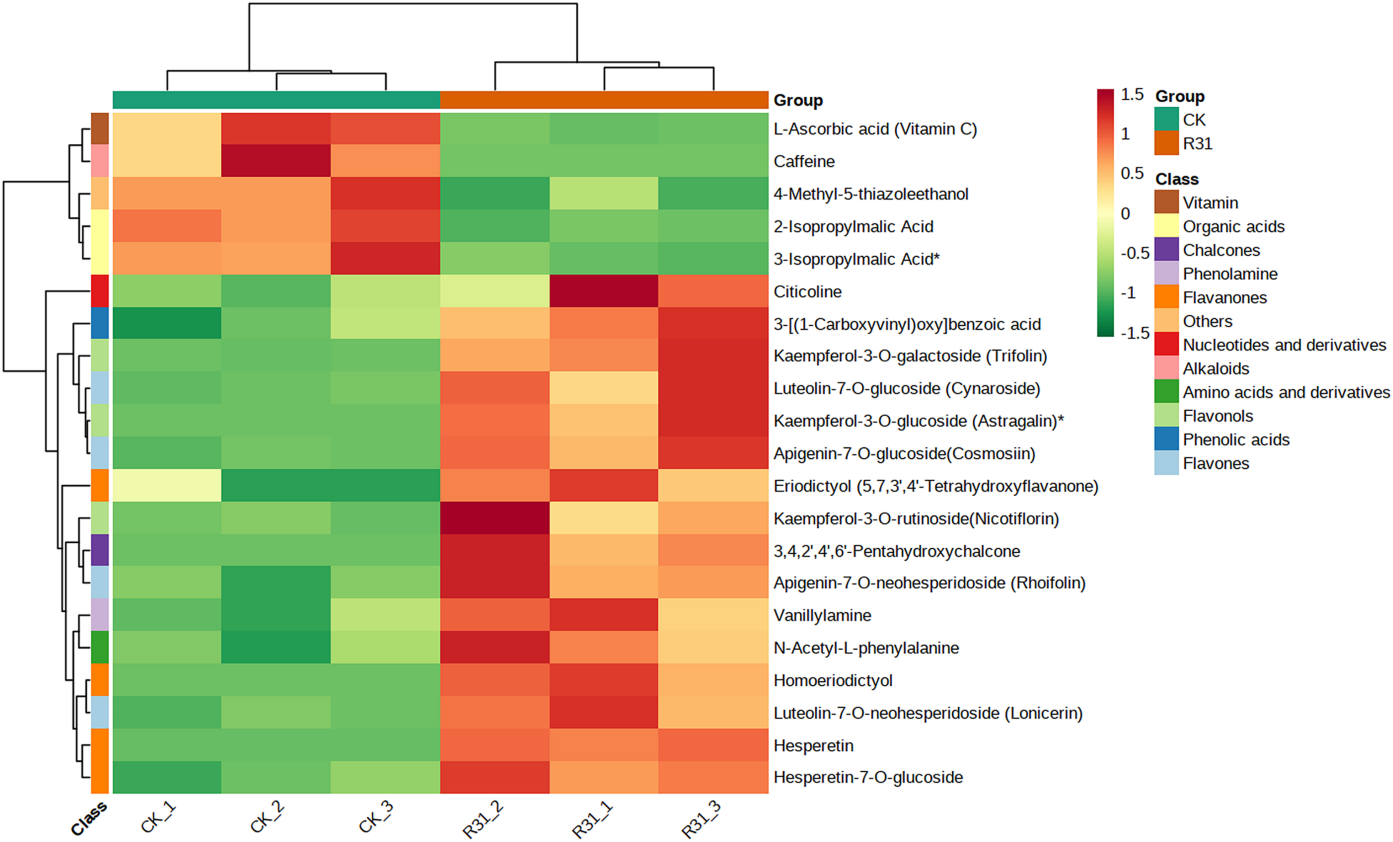
Heatmap of DAMs identified among sweet corn after application of *B. subtilis* R31. The horizontal coordinate is the name of the sample, and the ordinate is the differential metabolite. Different colors in the heat map represent the value obtained after the normalization of the relative content of the differential metabolite, reflecting the level of its relative content (red represents high content, green represents low content). The tree diagram on the left of the heat map represents the hierarchical clustering results of differential metabolites, and the comment bar on the right of the clustering tree corresponds to the first class classification of substances, with different colors representing different substance categories.

**Figure 7:**
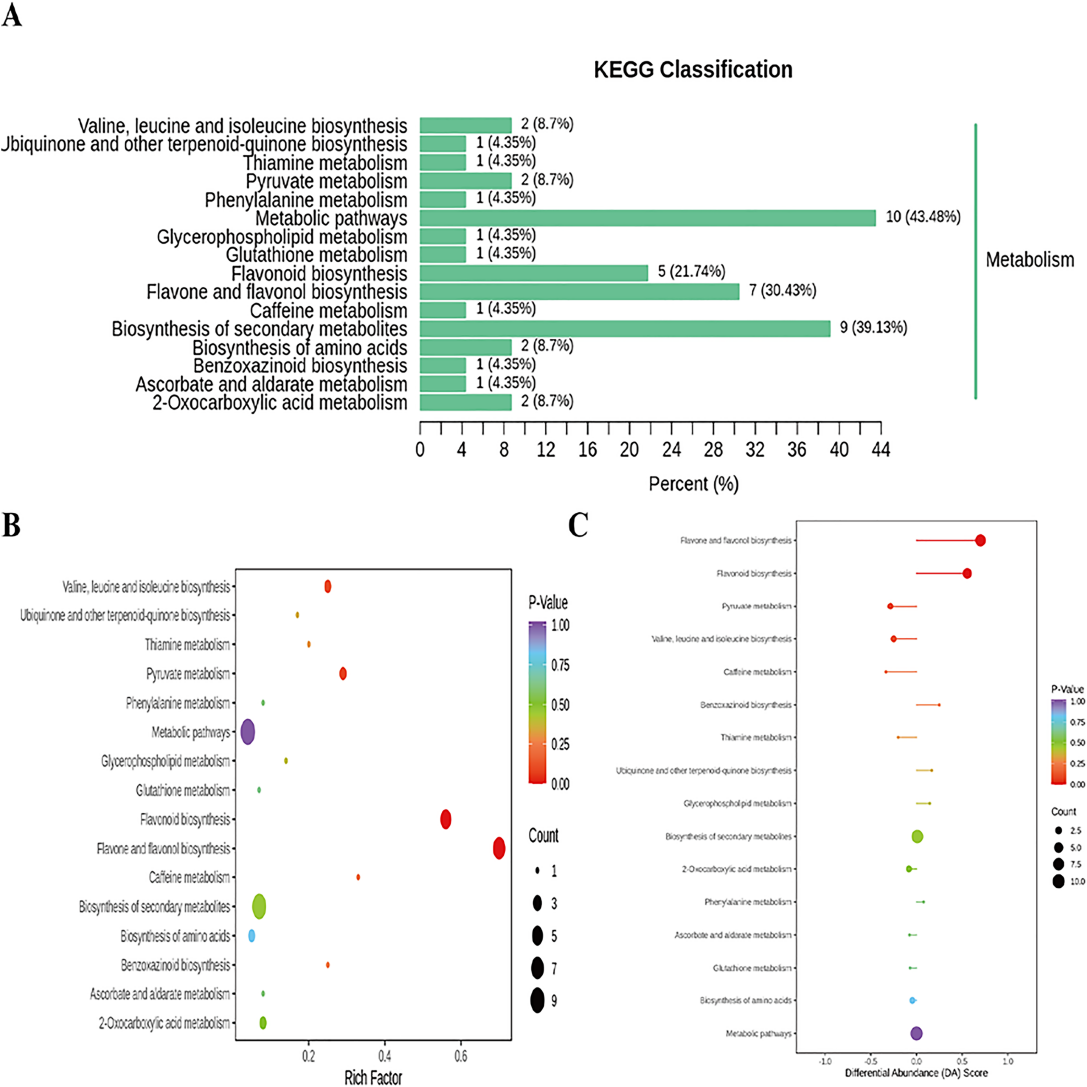
Enriched KEGG pathways of DAMs identified among sweet corn after application of *B. subtilis* R31. (A) KEGG classification map of differential metabolites. The ordinate represents the KEGG metabolic pathway, and horizontal coordinate represents metabolites annotated to the pathway and their proportion to the total number of annotated metabolites. (B) KEGG enrichment map of differential metabolites. The color of points reflects the *p*-value size, and the redder indicates the more significant enrichment. The size of the dot represents the number of enriched differential metabolites. (C) Plot of differential abundance score. The color of line segment and dot reflects the size of *p*-value.

In addition, cluster analysis of differential metabolites in KEGG signaling pathway results show that the upregulated DAMs were divided into citicoline, 3-[(1-carboxyvinyl)oxy] benzoic acid, kaempferol-3-O-galactoside (trifolin), luteolin-7-O-glucoside (cynaroside), kaempferol-3-O-glucoside (astragalin), apigenin-7-O-glucoside (cosmosiin), eriodictyol (5,7,3′,4′-tetrahydroxyflavanone), kaempferol-3-O-rutinoside (nicotiflorin), 3,4,2′,4′,6′-pentahydroxychalcone, apigenin-7-O-neohesperidoside (rhoifolin), vanillylamine, N-acetyl-L-phenylalanine, homoeriodictyol, luteolin-7-O-neohesperidoside (lonicerin), hesperetin, and hesperetin-7-O-glucoside pathways (Fig. 6 and Table S20). KEGG database analysis shows that DAMs were mainly classified into metabolic pathways (10), biosynthesis of secondary metabolites (nine), flavone and flavonol biosynthesis (seven), and flavonoid biosynthesis (five) (Fig. 7A). KEGG enrichment analysis indicates that metabolites involved in the pathways of “flavonoid biosynthesis” and “flavone and flavonol biosynthesis” showed the highest accumulation (Fig. 7B). Plot of differential abundance score (Fig. 7C) indicated that DAMs distributed in “flavonoid biosynthesis” and “flavone and flavonol biosynthesis” showed a relatively clear upward trend, which was consistent with the aforementioned transcriptomics analysis of gene expression. Flavonoids are polyphenolic compounds synthesized through the phenylpropanoid pathway. They play a multifunctional role in plant defense against environmental stresses, pathogens, herbivores, and UV-Bradiation (Li et al., 2021b).

## Discussion

Biocontrol bacteria have become the focus of research on the characteristics of environmental protection and plant disease control. So far, people have isolated and screened various types of biocontrol bacteria with different degrees of control effects on various plant diseases from soil and other environments, some of which have been used in agricultural production and have achieved good social and economic benefits. During our study, there were appreciable differences in sweet corn crop quality after application of *B. subtilis* R31, which indicates that the metabolites and genes associated with the treated sweet corn were altered by the biocontrol *B. subtilis* R31. Corn is the leading cereal and a good agricultural economic crop in China and the world and is ideally suited to the growth-promoting effect of biocontrol bacteria studies. Improvements in molecular and genomic technologies have allowed the analysis of physiological metabolism under different conditions and gene expression under different time series.

It is interesting to note that the pathways of “plant hormone signal transduction,” “plant-pathogen interaction,” “MAPK signaling pathway-plant,” “phenylpropanoid biosynthesis”, “glycolysis/gluconeogenesis”, and “tryptophan metabolism” showed enrichment, which was related to the plant’s defensive responses (Fig. 3B). Plants resist attacks by pathogens *via* two modes of innate immune response: pattern-triggered immunity (PTI) and effector-triggered immunity (ETI). PTI is triggered by microbial signatures, which are now called pathogen-or microbe-associated molecular patterns (PAMPs or MAMPs), whereas ETI is triggered by effectors secreted by host-adapted pathogens (Jones & Dangl, 2006). PTI and ETI work together to combat pathogen virulence, and they have many overlapping downstream responses, such as reactive oxygen species (ROS) bursts, MAPK activation, phytohormone signaling, defense gene expression, transcriptional reprogramming, and secondary metabolite production (Tsuda & Katagiri, 2010; Zhao et al., 2011; Piasecka, Jedrzejczak-Rey & Bednarek, 2015; Yu et al., 2017). MAPK cascades are important signaling modules in eukaryotes. A typical MAPK cascade consists of MAPKs/MPKs, MAPK kinases (MAPKKs/MKKs/MEKs), and MAPKK kinases (MAPKKKs/MKKKs/MEKKs). In plants, MAPK cascades play a fundamental role in cytokinesis, stomatal development, root growth, and embryonic patterning. In addition to its critical role in plant development, the MAPK cascade is also involved in plant immunity (Jones & Dangl, 2006). They function as molecular switches and transduce extracellular stimuli into intracellular signals to activate plant defense responses. In other words, the high expression of these genes may improve the resistance of sweet corn to biocontrol *B. subtilis*.

Many beneficial organisms can significantly affect plants, including promoting its growth and defense responses against pests and pathogens (Ogran et al., 2019; Arnaouteli et al., 2021; Fu et al., 2021). For example, trichoderma-treated tomato plants showed good anti-insect activity (Manganiello et al., 2018). Transcriptomic and metabolomic data indicate that the trichoderma contributes to the priming of overexpression of bZIP, MYB, NAC, AP2-ERF, and WRKY (defense-related transcription factors), which enhances both direct and indirect defense responses toward the aphid parasitoid *Aphidius ervi* (Coppola et al., 2019). In general, biological control has the basic characteristics of high efficiency, non-polluting, harmless, and non-toxic, which conform to the sustainable development of agriculture. At the same time, modern biotechnology provides a good opportunity for the development and expansion of biological control of plant diseases and a broad application prospect for microbial biological control. Moreover, the majority of upregulated secondary metabolites of sweet corn with biocontrol *B. subtilis* were glycosyl derivative of flavonoids, which are widely distributed secondary metabolites that play an important role in plant defense systems.

## Conclusions

In summary, this study is the first to report the quality characteristics, transcriptome, and metabolome of sweet corn influenced by the biocontrol *B. subtilis* R31. The results suggest that sweet corn was more fruitful and genes related to plant-pathogen interactions, MAPK signaling pathway-plant, phenylpropanoid biosynthesis, and flavonoid biosynthesis were significantly enriched after application of *B. subtilis* R31. The biocontrol of *B. subtilis* R31 appreciably improved the quality of sweet corn. Analysis of the metabolome and transcriptome showed that the metabolites and genes associated with the treated sweet corn were altered by the biocontrol *B. subtilis* R31. The detected DEGs related to the pathways of pyruvate metabolism, glycerolipid metabolism, fatty acid degradation, beta-alanine metabolism, ascorbate, aldarate metabolism, plant hormone signal transduction, plant-pathogen interaction, MAPK signaling pathway-plant, phenylpropanoid biosynthesis, and flavonoid biosynthesis suggests that biocontrol *B. subtilis* R31 improves the resistance of sweet corn. Moreover, the upregulated glycosyl derivative flavonoids may play an important role in human health. However, the specific mechanism by which the glycosyl derivative flavonoids are improved by *B. subtilis* R31 requires further elucidation. Overall, our study provides evidence that biocontrol bacteria improve crop quality and disease resistance, which may help to investigate the molecular mechanisms by which biocontrol bacteria enhance crop nutrition and taste.

## Supplemental Information

10.7717/peerj.14967/supp-1 Supplemental Information 1 Supplemental Tables.

10.7717/peerj.14967/supp-2 Supplemental Information 2 Supplemental Figures.

10.7717/peerj.14967/supp-3 Supplemental Information 3 Raw data.

## Additional Information and Declarations

### Data Availability

The following information was supplied regarding data availability: The transcriptome of sweet corn is available at NCBI SRA BioProject: PRJNA852671.
