# Supplementary material for: Biocontrol endophytes Bacillus subtilis R31 influence the quality, transcriptome and metabolome of sweet corn
Source: PeerJ. 2023 Mar 2;11:e14967. doi: 10.7717/peerj.14967 (PMC9985898; doi:10.7717/peerj.14967)
Supplement: Supplemental Information 2 [file peerj-11-14967-s002.docx]

**Supporting Information**

**Biocontrol Endophytes *Bacillus subtilis* R31 Influence the Quality, Transcriptome and Metabolome of Sweet Corn**

**Guidance – remove this box before submitting!**

Yellow callout boxes provide general notes. Please remove the yellow boxes before submitting. For full guidance see <https://peerj.com/about/author-instructions>

Blue highlighted example text should be replaced or removed with your own information.

| **DO**  –Use clear and grammatically correct English.  –Save as US Letter size format.  –Ensure line numbering is enabled.  –Align text LEFT.  –Ensure title, abstract, and author information matches what is entered online during submission. | **DO NOT**  –Embed ANY figures or tables in the text. Instead, upload a separate file for each on the file uploads page when submitting. Example – If you have 3 figures, then you will upload 3 figure files & be asked to add a figure title for each. See <https://peerj.com/about/author-instructions/#figures> for figure formats. |
| --- | --- |

Mingwei Shao ^1, 2, 3, 4,^ *, Yanhong Chen ^5,^ * , Qingyou Gong ^5^, Shuang Miao ^1, 2, 3, 4^, Chunji Li ^1, 2, 3, 4^, Yunhao Sun^1, 2, 3, 4^, Di Qin ^1, 2, 3, 4^, Xiaojian Guo ^1, 2, 3, 4^, Xun Yan ^1, 2, 3, 4^, Kai Liu^1, 2, 3, 4^, Ping Cheng ^1,^ ^2, 3, 4^* and Guohui Yu ^1, 2, 3,4^

^1^Innovative Institute for Plant Health, Zhongkai University of Agriculture and Engineering, Guangzhou 510225, People’s Republic of China

^2^College of Agriculture and Biology, Zhongkai University of Agriculture and Engineering, Guangzhou 510225, People’s Republic of China

^3^Key Laboratory of Green Prevention and Control on Fruits and Vegetables in South China, Ministry of Agriculture and Rural Affairs, People’s Republic of China, Guanghzou, 510225, People’s Republic of China

^4^Guangdong University Key Laboratory for Sustainable Control of Fruit and Vegetable Diseases and Pests, Guangzhou, 510225, People’s Republic of China

^5^Zhuhai Modern Agriculture Development Center, Zhuhai 519075, People’s Republic of China

Corresponding Author:

Ping Cheng ^1, 2, 3, 4^, Guohui Yu ^1, 2, 3, 4^

No. 24, Dongsha Street Guangzhou 510225, China

Email address: 542267567@qq.com; ygh76411@zhku.edu.cn

**Supporting Information Table of Contents**

| **Description of Supporting Item** | **Page** |
| --- | --- |
| **Figure S1.** After assembling the clean reads, there are a alignment efficiency more than 84.0%. | **S3** |
| **Figure S2.** The proportion of read distribution located in exon regional is more than 86.0%. | **S4** |
| **Figure S3.** The unigenes of sweet corn after application of *B. subtilis* R31  annotated in NR (A) and eggNOG (B) databases. | **S5** |
| **Figure S4**. The unigenes annotated in the eggNOG database. | **S6** |
| **Figure S5.** Correlation evaluation of biological repetition for transcriptome analyses of sweet corn after application of *B. subtilis* R31. | **S7** |
| **Figure S6.** Heatmap of DEGs expression in sweet corn after application of *B. subtilis* R31. | **S8** |
| **Figure S7.** Classification map of secondary entries of DEGs expression in sweet corn after application of *B. subtilis* R31. | **S9** |
| **Figure S8.** Histogram of GO enrichment for top50 DEGs expression in sweet corn after application of *B. subtilis* R31. | **S10** |
| **Figure S9**. KOG classification bar chart of DEGs expression in sweet corn  after application of *B. subtilis* R31. | **S11** |
| **Figure S10.** OPLS-DA of DAMs identified among sweet corn after  application of *B. subtilis* R31. (A) OPLS-DA score plot. (B) PLS-DA score  plot. | **S12** |
| **Figure S11.** Top Fc distribution of DEGs expression in sweet corn after application of *B. subtilis* R31. | **S13** |


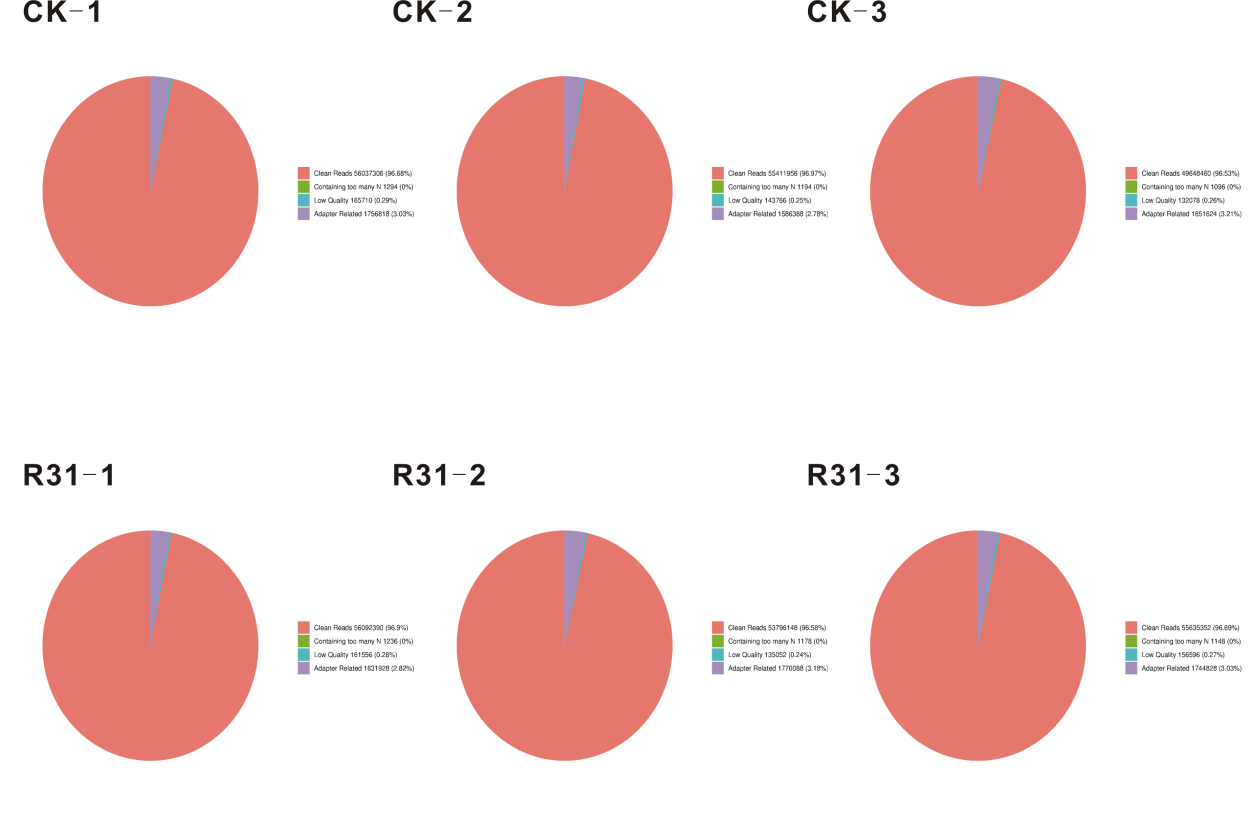


Figure S1. After assembling the clean reads, there are a alignment efficiency more than 84.0%.


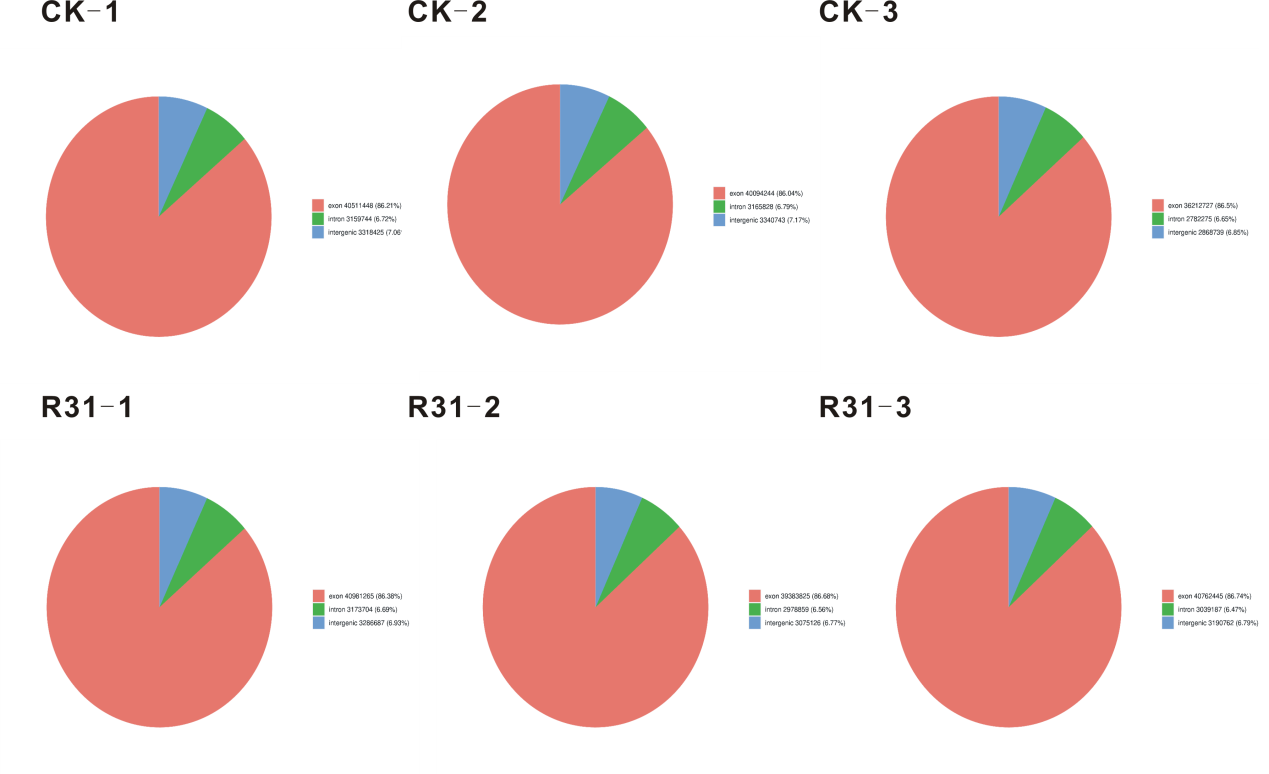


Figure S2. The proportion of read distribution located in exon regional is more than 86.0%.


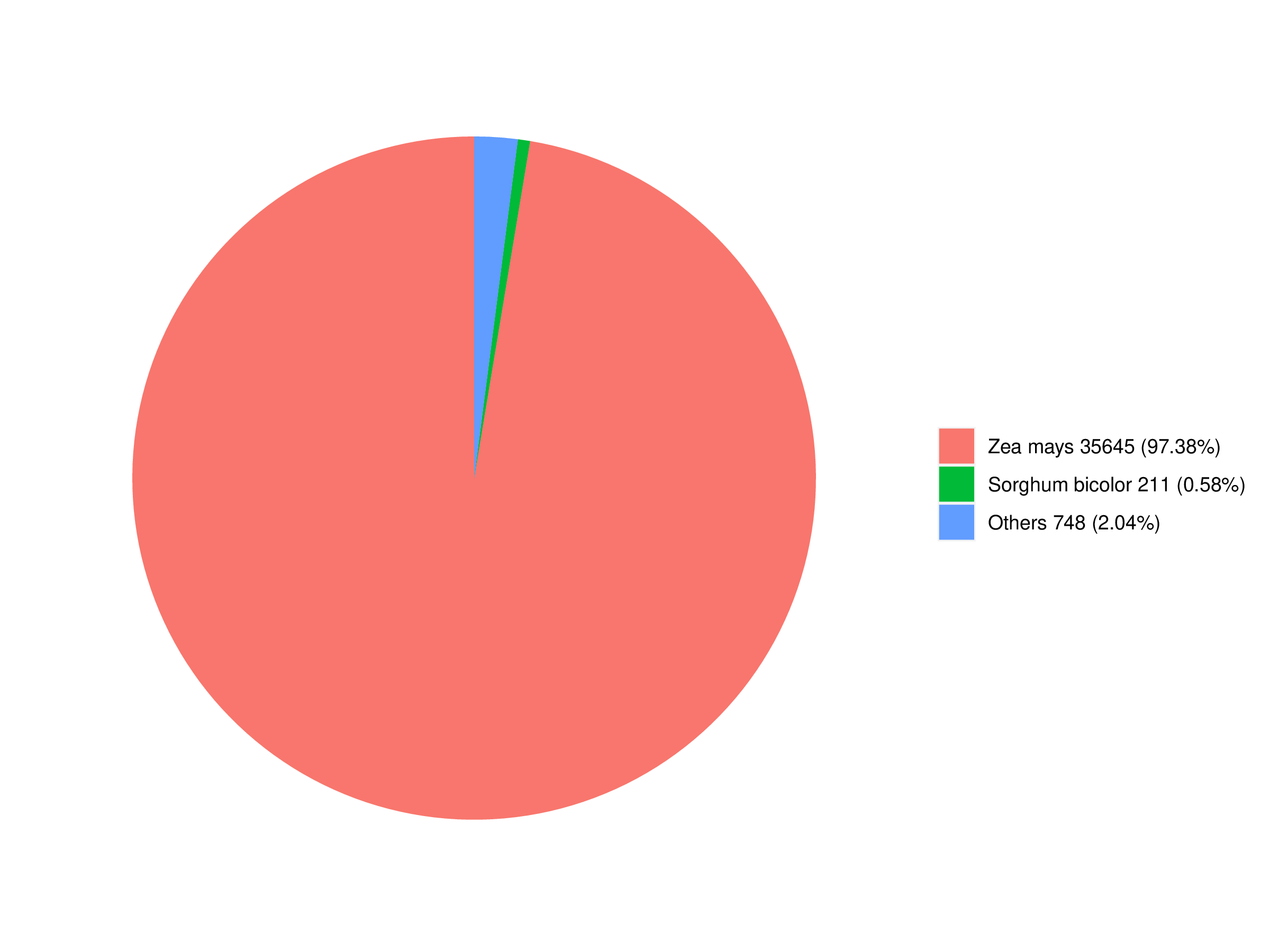


Figure S3. The unigenes of sweet corn after application of *B. subtilis* R31 annotated in NR (A) and eggNOG (B) databases.


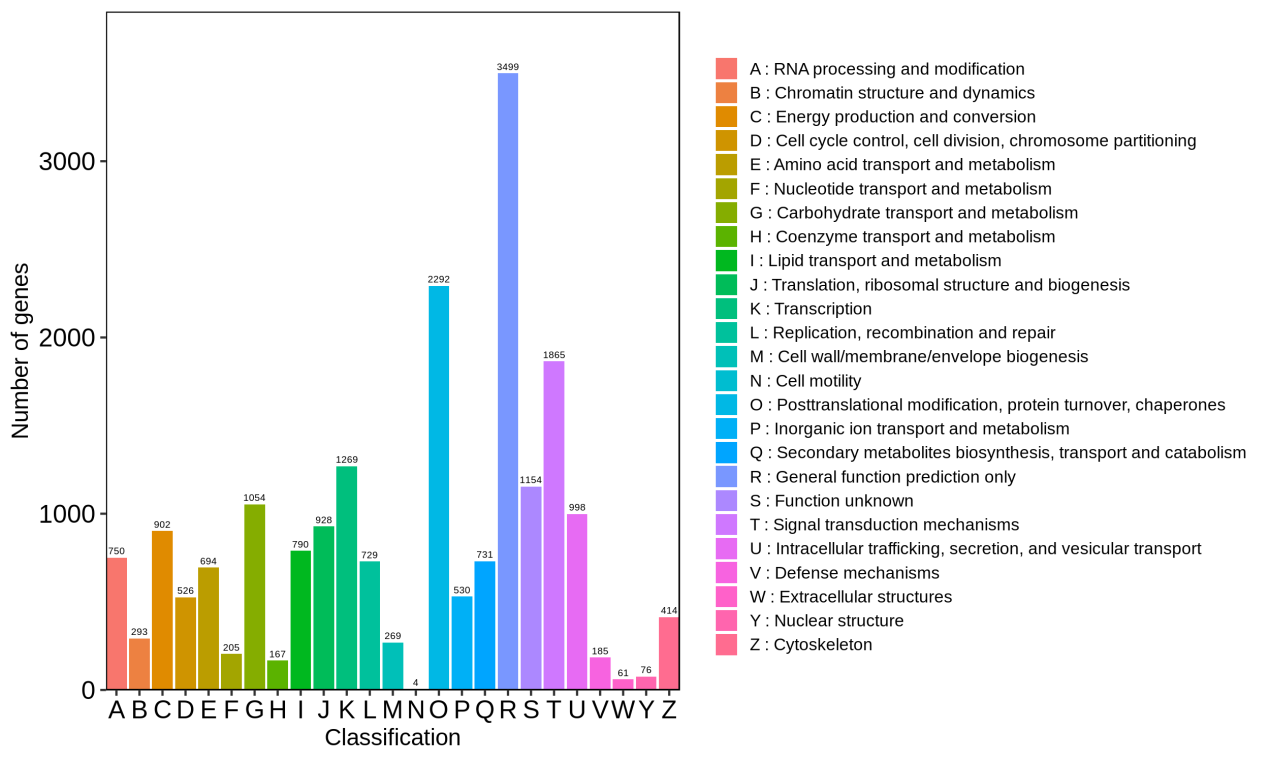


Figure S4. The unigenes annotated in the eggNOG database.


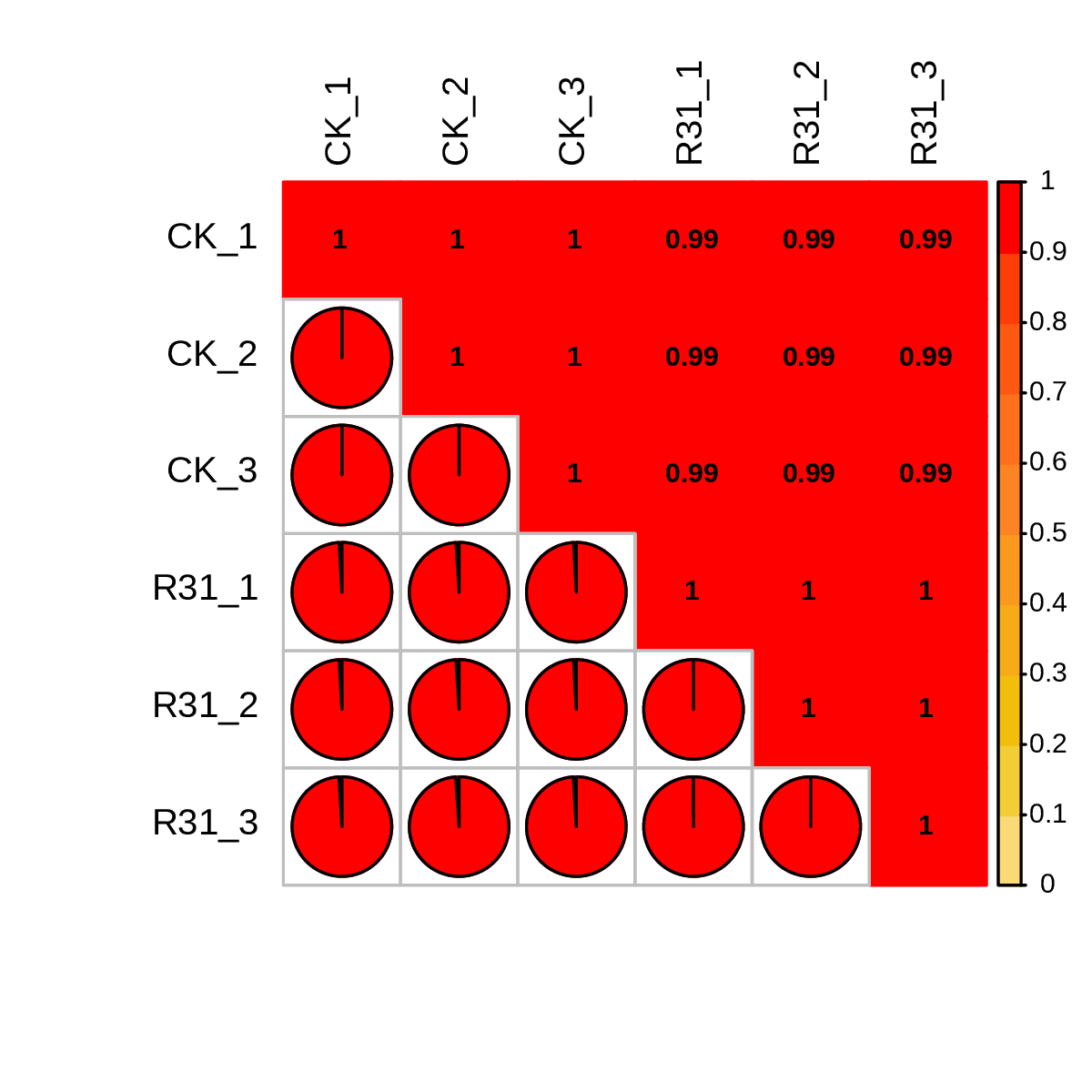


**Figure S5.** Correlation evaluation of biological repetition for transcriptome analyses of sweet corn after application of *B. subtilis* R31.


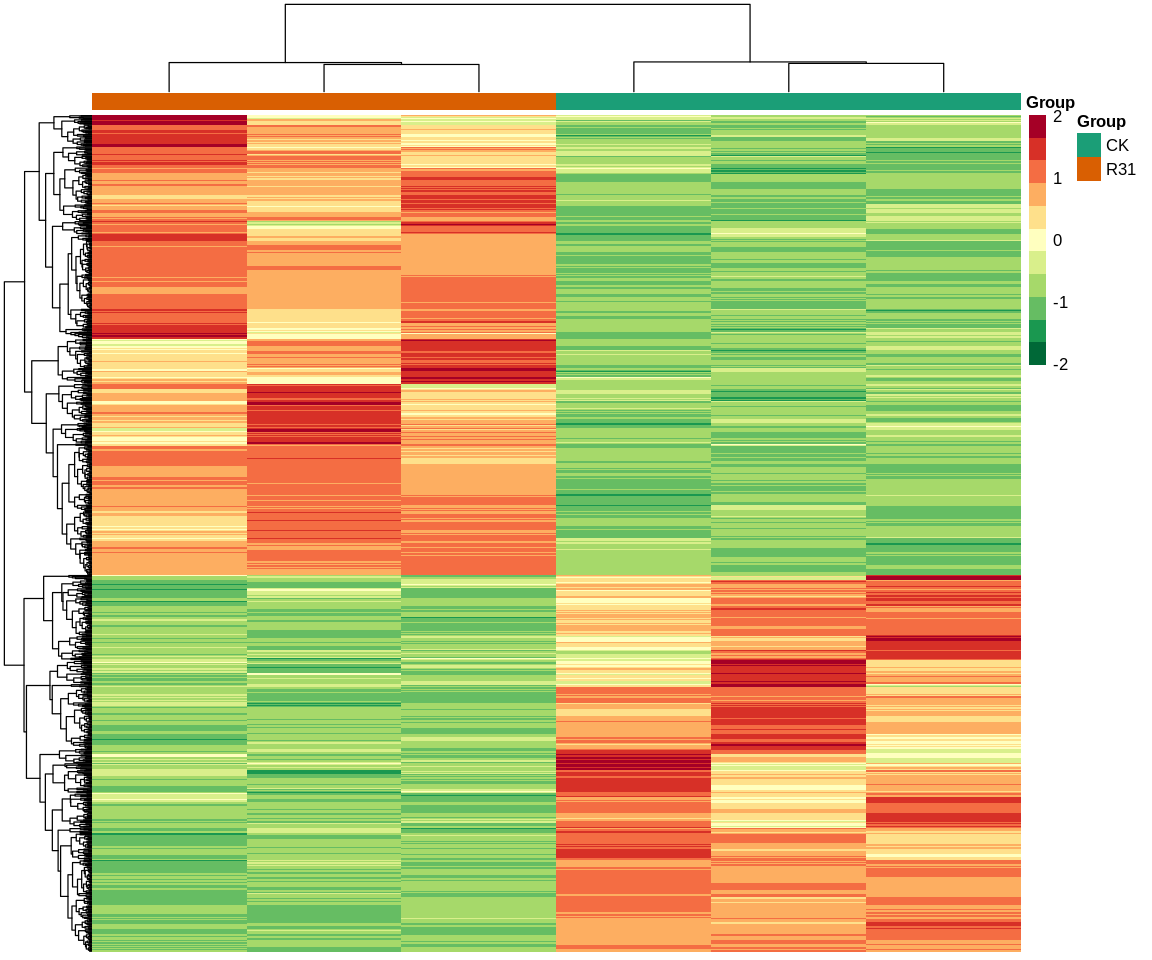


**Figure S6.** Heatmap of DEGs expression in sweet corn after application of *B. subtilis* R31. Horizontal is sample name, vertical is metabolite information, group is grouping, class is substance classification, and different colors are values obtained after standardized treatment of relative content (red represents high content, green represents low content).


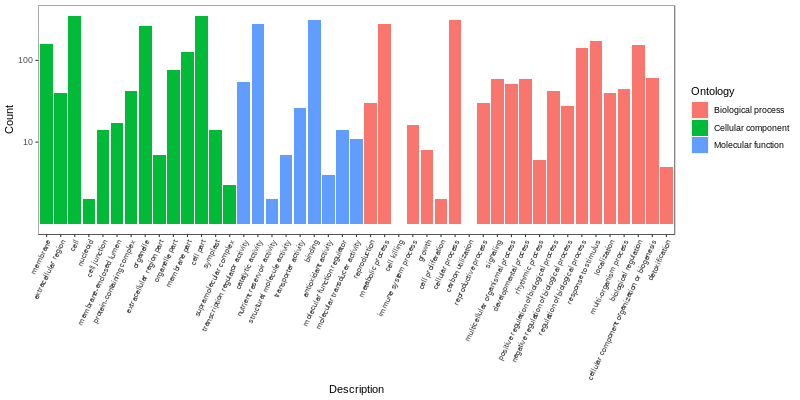


**Figure S7**. Classification map of secondary entries of DEGs expression in sweet corn after application of *B. subtilis* R31. The horizontal coordinate represents the secondary GO entries, and the vertical coordinate represents the number of differential genes in the GO entries (Green column represents biological process, blue column represents cellular compound, and orange column represents molecular function).

**
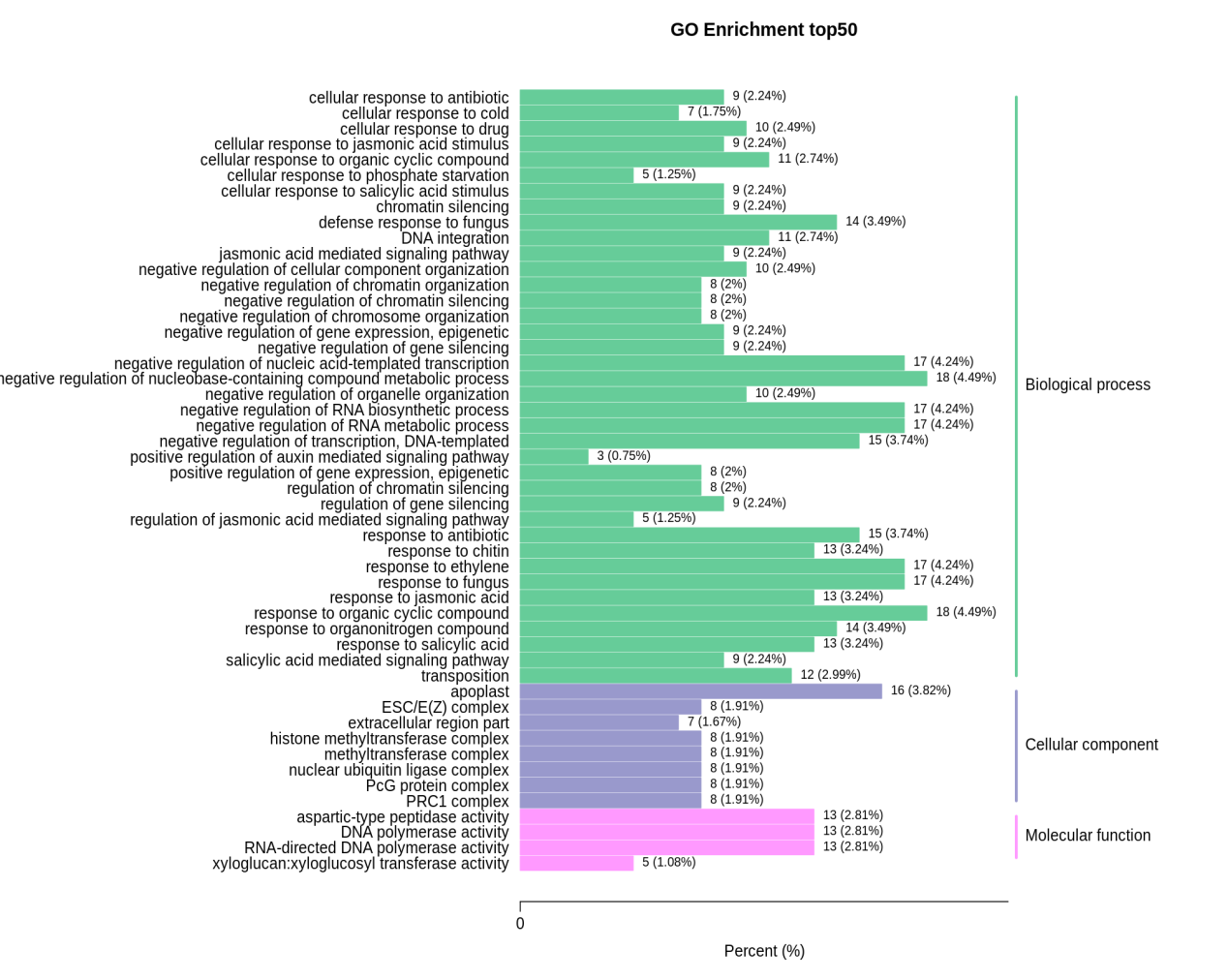
**

**Figure S8.** Histogram of GO enrichment for top50 DEGs expression in sweet corn after application of *B. subtilis* R31. The horizontal coordinate represents the ratio of commented genes to the total number of annotated genes, and the vertical coordinate represents the name of the GO entry. The label to the right of the graph represents the category to which the GO entry belongs (Green column represents biological process, lilac column represents cellular compound, and pink column represents molecular function).


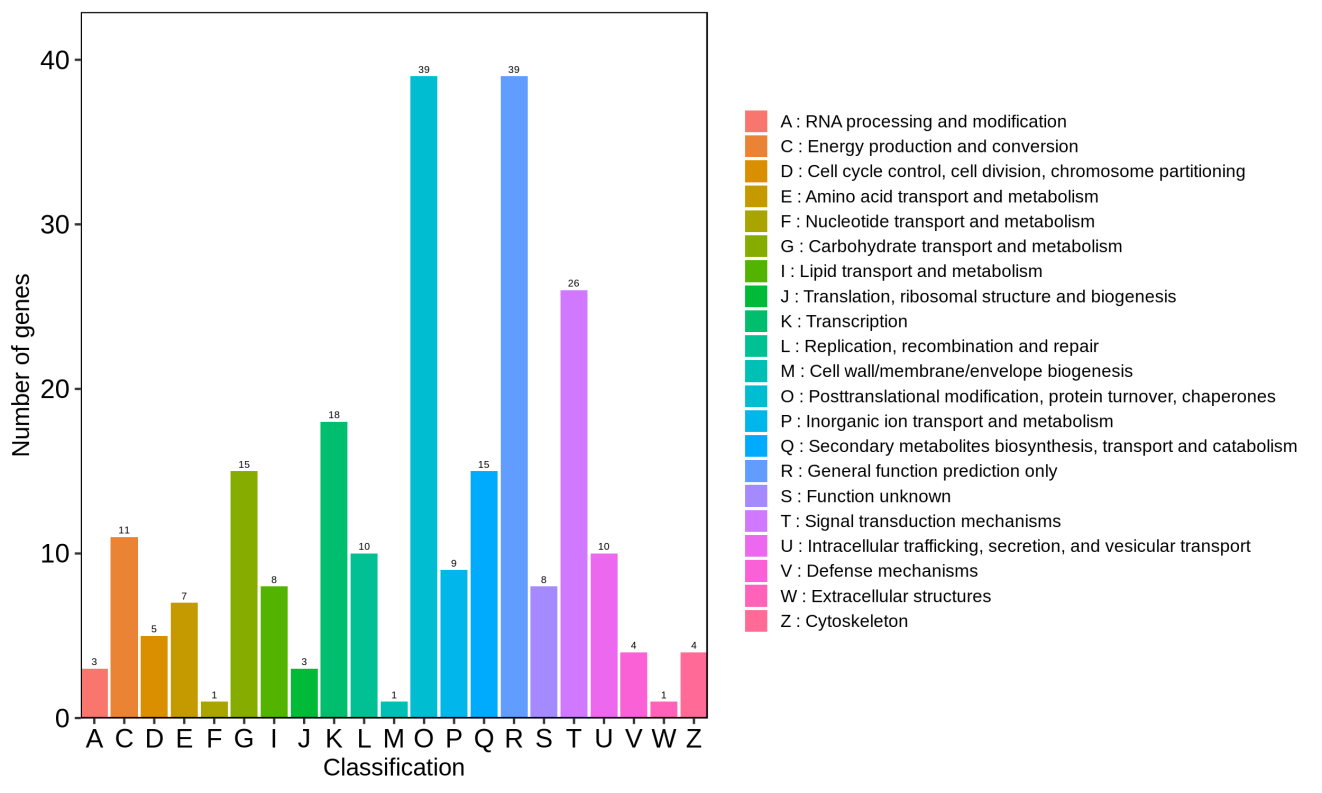


**Figure S9.** KOG classification bar chart of DEGs expression in sweet corn after application of *B. subtilis* R31.


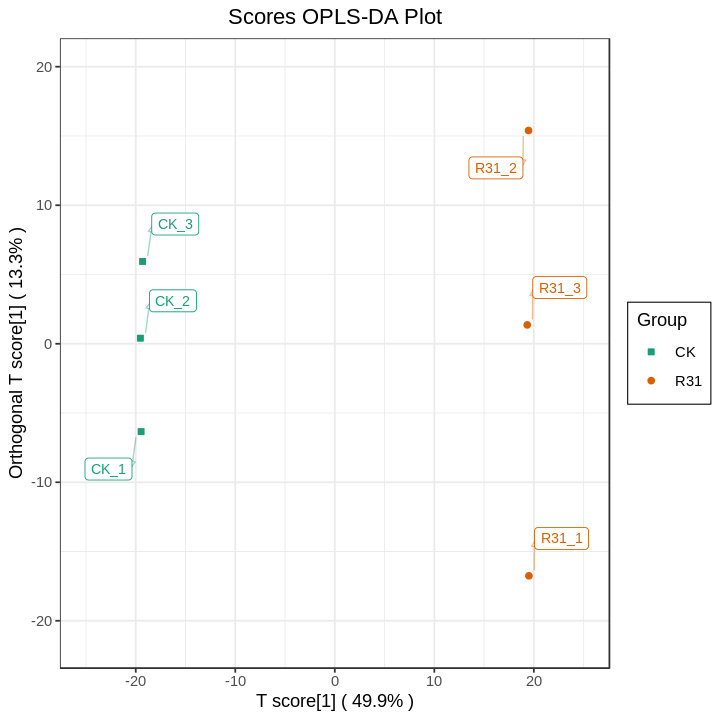

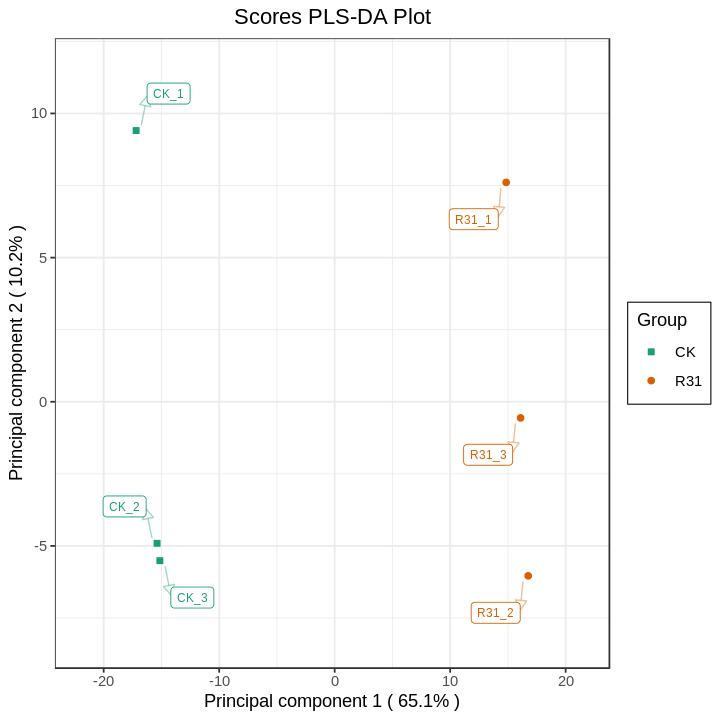


**Figure S10.** OPLS-DA of DAMs identified among sweet corn after application of *B. subtilis* R31. (A) OPLS-DA score plot. (B) PLS-DA score plot.


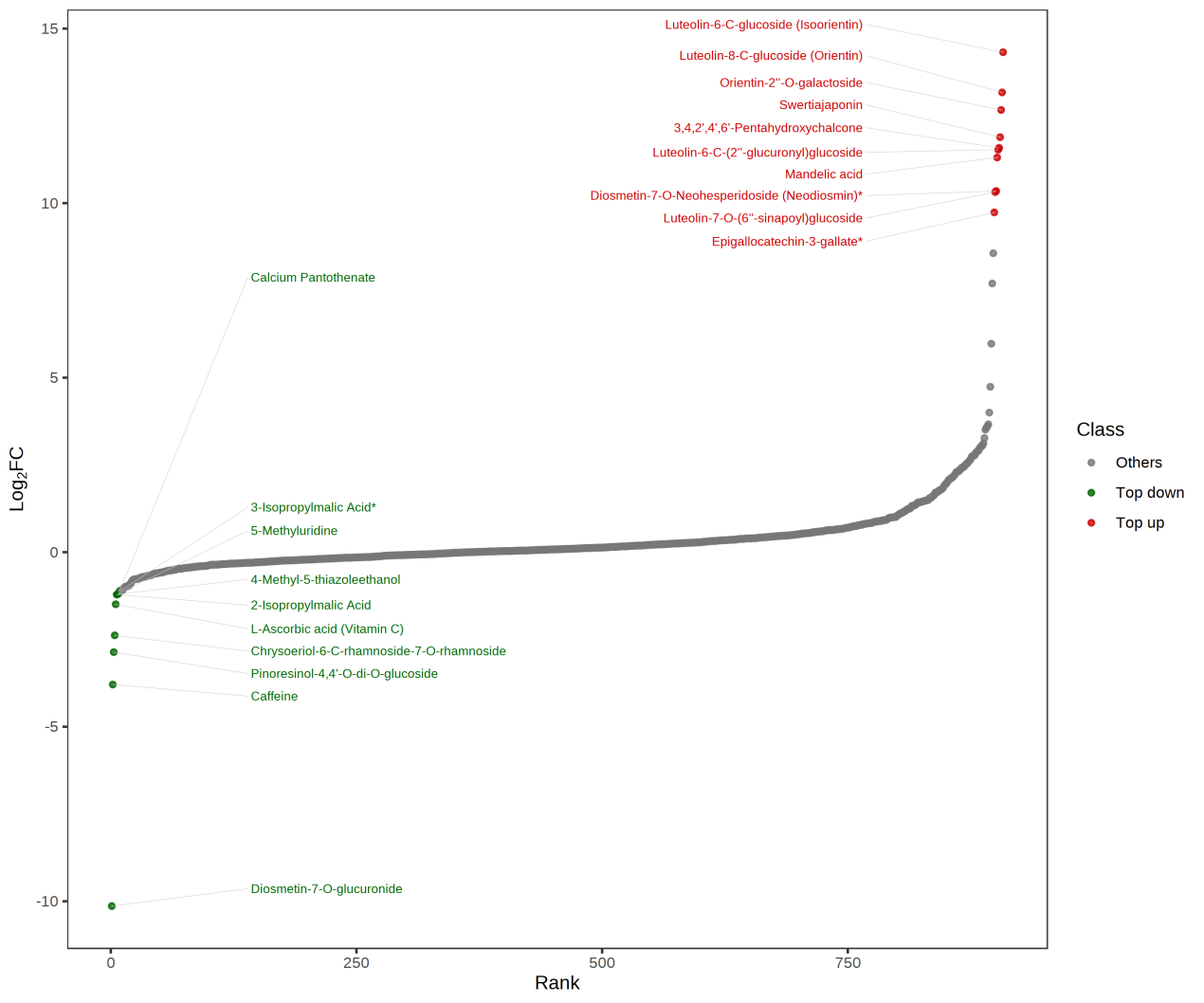


**Figure S11** Top Fc distribution of DEGs expression in sweet corn after application of *B. subtilis* R31. The horizontal axis represents VIP value, and the vertical axis represents differential metabolites. The red dot represents up-regulated differential metabolites, and the green dot represents down-regulated differential metabolites.
